# Outstanding treatment success of pembrolizumab and bevacizumab combination therapy in first-line treatment of cervical sarcomatoid carcinoma: a rare case report

**DOI:** 10.3389/fonc.2025.1586531

**Published:** 2025-09-08

**Authors:** Mingzi Zhang, Xinbei Li, Zhonghua Huang, Zhenjiang Yang, Lina Yang

**Affiliations:** ^1^ Department of Pharmacy, Shenzhen Traditional Chinese Medicine Hospital, Shenzhen, China; ^2^ Department of Radiology, Shenzhen Traditional Chinese Medicine Hospital, Shenzhen, China; ^3^ Department of Pathology, Shenzhen Traditional Chinese Medicine Hospital, Shenzhen, China; ^4^ Department of Internal Medicine of Oncology and Hematology, Shenzhen Traditional Chinese Medicine Hospital, Shenzhen, China

**Keywords:** sarcomatoid carcinoma, cervix, pembrolizumab, bevacizumab, PD-L1

## Abstract

**Background:**

Cervical lesions of sarcomatoid carcinoma are very rare, and there are still no reports of any targeted drugs applied in this rare tumor. In this report, we document a patient with stage IVA sarcomatoid carcinoma of the cervix, according to the FIGO staging system. The genetic testing of the patient’s tumor tissue indicated the expression of PD-L1. This finding is significant as it suggests that the patient may be a candidate for immunotherapy. In this manuscript, we report a case of a patient who achieved a transient recurrence-free survival period through combined therapy (although recurrence eventually occurred), with a progression-free survival exceeding 13 months and an overall survival exceeding 22 months (as of the last follow-up, the patient was receiving palliative care only).

**Case presentation:**

After diagnostic confirmation, the patient was administered a first-line combination therapy consisting of pembrolizumab plus bevacizumab. After 2 cycles of treatment, there was a marked reduction in tumor volume and the patient did not experience any side effects. Since then, the patient has continued to receive the regimen and the tumor has continued to shrink. Ultimately, after 10 courses of treatment with immune checkpoint inhibitors and anti-angiogenic drugs, the PET-CT scan showed complete disappearance of the tumor, with no evidence of cancer throughout the body. Subsequently, the patient continued to receive maintenance therapy with the same regimen, with regular follow-up evaluations. No recurrence was detected until 13 months later, when a MRI scan revealed tumor recurrence.

**Conclusions:**

The combination of a PD-1 inhibitor with a drug that promotes tumor vascular normalization has shown promise in treating advanced cervical sarcomatoid carcinoma. We are the first to report the use of this combination regimen in this rare tumor, where previously reported treatment has been with chemotherapy agents. In addition, the level of PD-L1 expression could serve as a potential biomarker to predict the response to immunotherapy in patients with advanced sarcomatoid carcinoma of the cervix. Our case highlights the efficacy of immunotherapy in combination with anti-angiogenic targeted therapy for the treatment of sarcomatoid carcinoma of the cervix.

## 1 Introduction

The entity of sarcomatoid carcinoma is seldom encountered in the pathology of the uterine cervix (1, 2). Sarcomatoid carcinoma of the cervix (SCC) has both epithelial and sarcomatoid stromal tissue components, sarcomatoid tissue is usually dominant, squamous cell carcinoma is the main epithelial component (3, 4). Although the classification system of World Health Organization (WHO) for gynecological tumors does not formally acknowledge sarcomatoid carcinoma as a separate histological subtype of cervical cancer, it is noteworthy that this rare malignancy has been reported in a few case studies in the medical literature (5). These reports provide valuable insights into the clinical presentation, management challenges, and outcomes associated with this rare and aggressive form of cervical cancer.

Patients typically exhibit symptoms at later stages and experience a highly aggressive progression of the disease. Due to the rarity of the cervical sarcomatoid carcinoma, there is no standardized diagnostic and therapeutic protocol. The majority of these cases are managed as squamous cell carcinoma and treated with either surgery or radiotherapy or chemotherapy (1, 6, 7). Although a significant therapeutic response to the initial treatment is observed in the majority of cases, subsequent relapses tend to occur after a short period (2, 3, 6). SCC is an exceptionally rare malignancy, with fewer than 40 cases documented in the existing medical literature, resulting in a scarcity of long-term follow-up data. Currently, the landscape of treatment for SCC is characterized by a variety of approaches, including surgery, radiotherapy, and chemotherapy (1). SCC generally has a poor prognosis, especially in advanced stages. Therefore, there is an urgent need to explore new and reliable therapeutic options. However, there is a notable absence of a standardized therapeutic protocol, as well as a lack of a systematic treatment plan tailored to this rare condition. SCC is recognized for its heightened invasiveness compared to conventional cervical carcinomas, characterized by a propensity for rapid progression, a tendency to relapse shortly after treatment, and a generally poor response to pre-existing therapeutic interventions (6).

Historically, sarcomatoid carcinoma has demonstrated a lack of sensitivity to conventional radiotherapy and chemotherapy, which has posed significant challenges in its management (8). Given these factors, the mainstay of treatment has centered on early diagnosis and the complete surgical resection of the tumor (7). Despite its sarcomatous features, sarcomatoid carcinoma retains epithelial origins, which means that traditional chemotherapeutic agents may still be utilized in an adjuvant capacity to complement surgical intervention (1). However, for patients presenting with advanced stages or those experiencing recurrence, the effectiveness of current chemotherapy options remains limited, and no definitively effective regimen has been reported to date. Given these challenges, there is a pressing requirement for the innovation of new therapeutic agents or specially designed alternative treatment strategies.

There are currently no clinical studies to assess the combination of immune checkpoint inhibitors (ICIs) with anti-angiogenic therapies for the treatment of programmed death-ligand 1-positive (PD-L1-positive) SCC. Pembrolizumab is a type of immune checkpoint inhibitor (ICI) that functions by blocking the programmed cell death protein 1 (PD-1) on T cells, allowing them to recognize and attack cancer cells (9). Bevacizumab is a type of monoclonal antibody designed to target vascular endothelial growth factor (VEGF), a protein that is crucial for the development of blood vessels that nourish tumors. By inhibiting VEGF, bevacizumab not only exerts anti-angiogenic effects but also induces transient vascular normalization in tumor vasculature, thereby limiting its growth and spread (10). This paper reports a case study that analyses the efficacy and safety profile of the combination treatment using pembrolizumab, an ICI, together with bevacizumab, an anti-angiogenic agent, for an advanced case of PD-L1-positive SCC.

## 2 Case presentation

**Figure 1:**
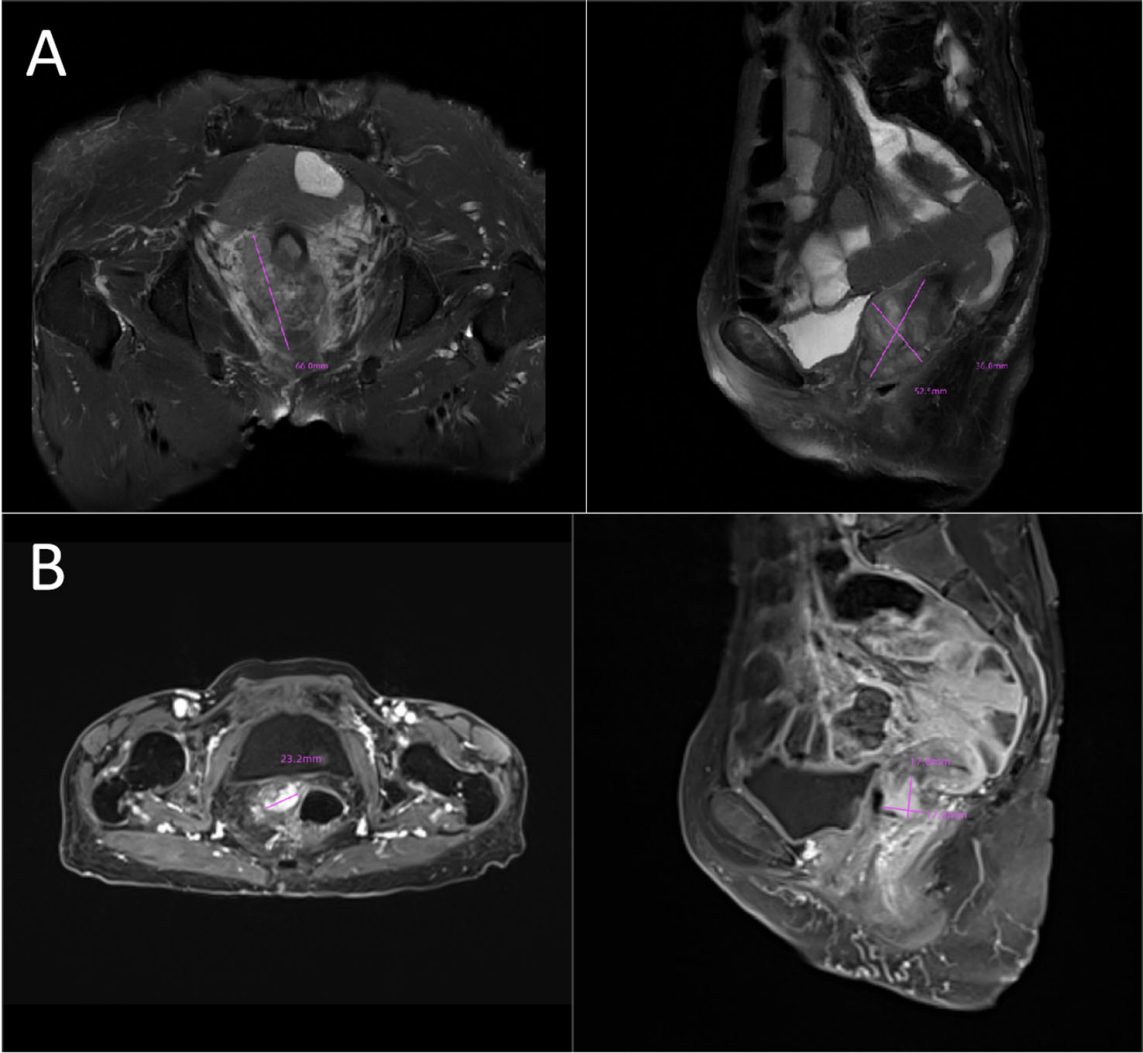
**(A)** Initial tumor mass:MRI image showing an occupying lesion in the anterior lip of the cervix, which is considered to be sarcomatoid carcinoma of the cervix (66mmx52.5mmx36mm). The boundary with the anterior wall of the rectum and the posterior wall of the bladder is unclear (FIGO stage IVA). **(B)** Post-treatment shrinkage:The pelvic MRI results showed a significant reduction in the size of the patient’s tumor (23.2mmx17.8mmx17.0mm). MRI, magnetic resonance imaging.

**Figure 2:**
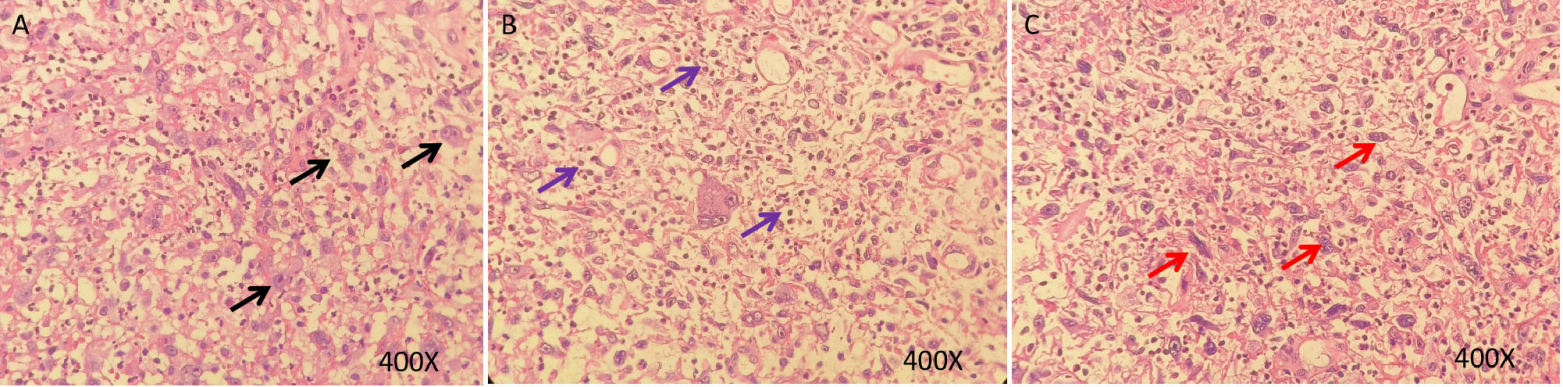
HE staining of sarcomatoid carcinoma of the cervix. **(A–C)**, H&E stain, original magnification ×400. HE staining of tumor showing a poorly differentiated malignant tumor with marked cellular atypia. The tumor cells exhibit epithelioid and myofibroblastic patterns, with abundant cytoplasm and prominent nucleoli. The background is mixed with granulation tissue and there is significant infiltration of inflammatory cells (Black arrows: Epithelioid carcinoma cells; Red arrows: Myofibroblast-like tumor cells; Purple arrows: Inflammatory infiltrates).

**Figure 3:**
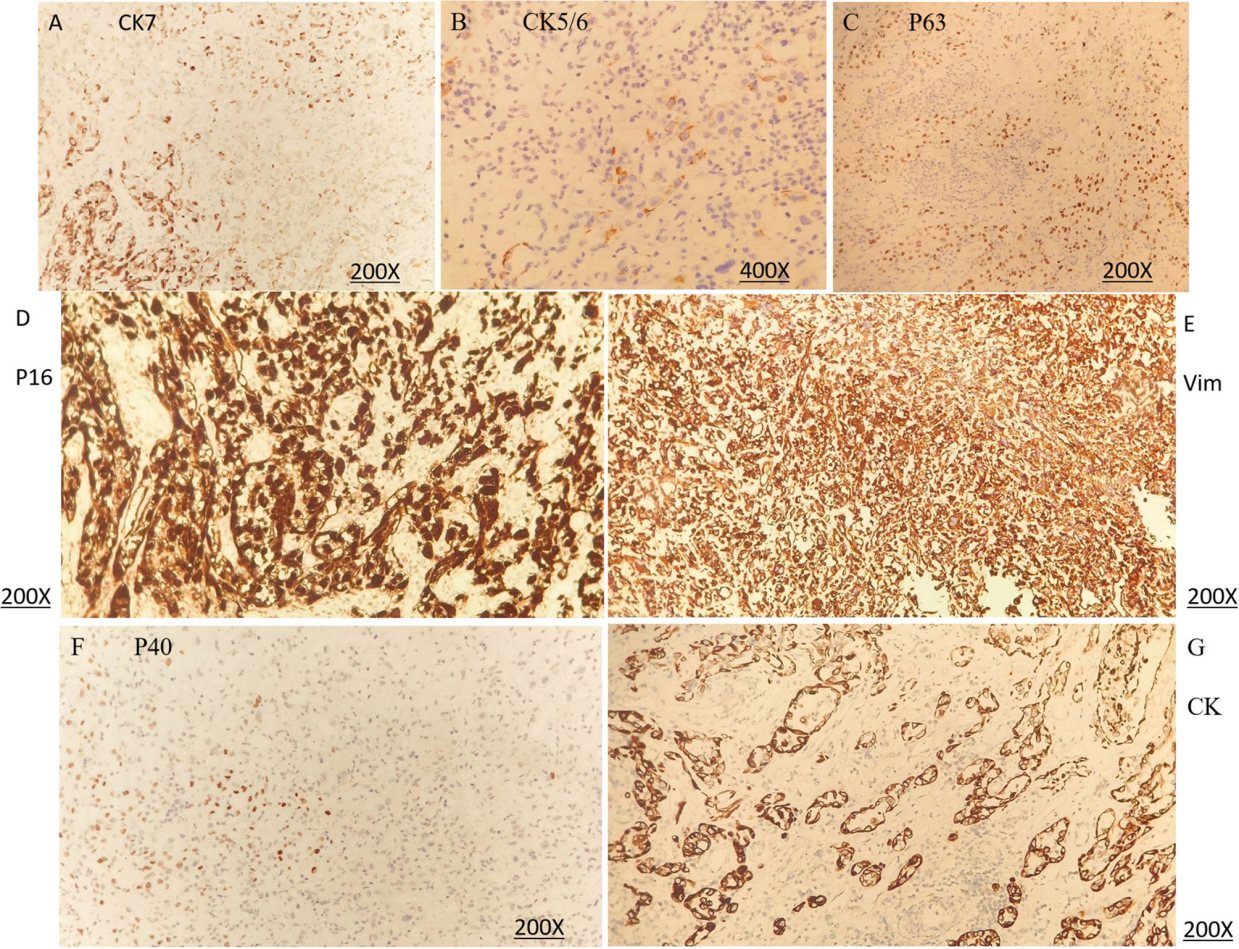
Immunohistochemical (IHC) staining of sarcomatoid carcinoma of the cervix. **(A)** CK7 (positivity), original magnification ×200. **(B)** CK5/6 (very rare positivity), original magnification ×400. **(C)** P63 (sparse positivity), original magnification ×200. **(D)** P16 (positivity), original magnification ×200. **(E)** Vimentin (positivity), original magnification ×200. **(F)** P40 (focal positivity), original magnification ×200. **(G)** CK (positivity, epithelioid component), original magnification ×200. **(A–F)** demonstrate protein expression in the sarcomatoid component, whereas **(G)** shows protein expression in the epithelioid component.

In 2023, a 73-year-old Chinese woman with a prior HPV-DNA test result positive for low-risk HPV type 70 presented to the Department of Internal Medicine of Oncology and Hematology at our hospital. She had been diagnosed with SCC at a local hospital approximately two weeks prior. The patient was in search of alternative treatment options, as she had declined the conventional approaches of radiotherapy and chemotherapy. The patient was admitted to a local hospital due to over 10 days of irregular vaginal bleeding without an immediately identifiable cause. Then, the patient underwent a pelvic magnetic resonance imaging (MRI) scan, which revealed an occupying mass in the anterior lip of the cervix, suggestive of cervical cancer, measuring 66 mm x 52.5 mm x 36 mm. The boundary with the anterior wall of the rectum and the posterior wall of the bladder is unclear (International Federation of Gynecology and Obstetrics (FIGO) stage IV4A) (**Figure 1A**). The patient subsequently underwent a colposcopic biopsy with hematoxylin and eosin (HE) staining and immunohistochemistry (IHC). The results of these tests were indicative of a predisposition to sarcomatoid carcinoma, leading to the diagnosis of SCC. The sarcomatoid component, as determined by comprehensive histopathological evaluation, accounted for 70% of the total tumor volume in this case (**Figures 2**, **3**). The patient, who was not in favor of undergoing chemotherapy and radiotherapy, decided to seek alternative options and turned to our hospital for further assistance and potential treatment avenues.

**Figure 4:**
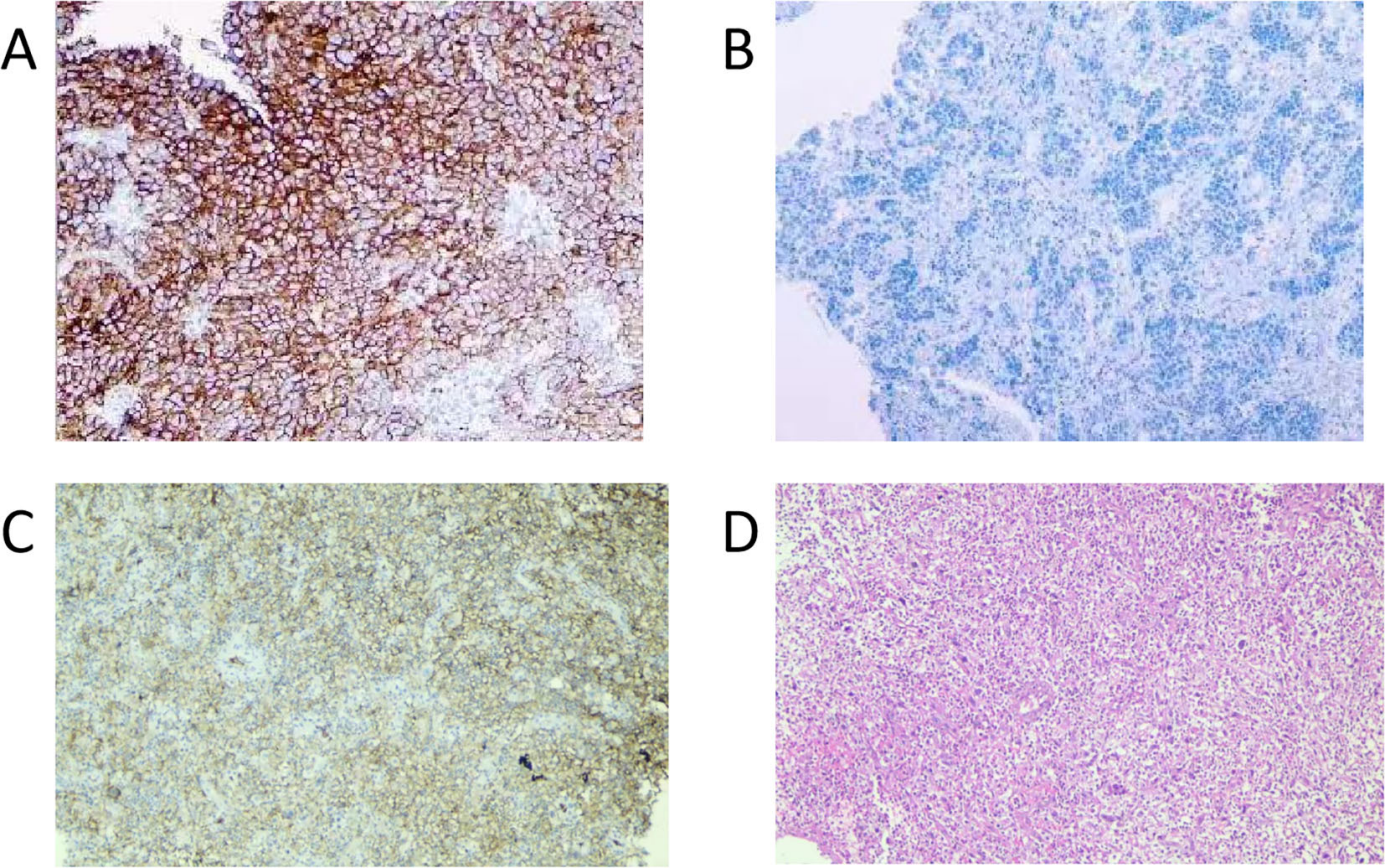
PD-L1 IHC of the tumor tissue. **(A)**, positive control (PD-L1-positive non-small cell lung cancer tissue), original magnification ×100. **(B)**, negative control (PD-L1-negative non-small cell lung cancer tissue), original magnification ×100. **(C)**, PD-L1 IHC of tissue from the patient in this case report (antibody 22C3 pharmDx), original magnification ×100. **(D)**, HE staining of tissue from the patient in this case report, original magnification ×100. Tumor Proportion Score (a TPS): 40% and Combined Positive Score (b CPS): 45. (a TPS is the percentage of living tumor cells that have partial or full PD-L1 membrane staining, assessed in a sample of at least 100 living tumor cells. b CPS is calculated by dividing the count of PD-L1 positive cells (such as tumor cells, lymphocytes, and macrophages) by the total tumor cell count, then multiplying the quotient by 100 to express it as a percentage.

**Figure 5:**
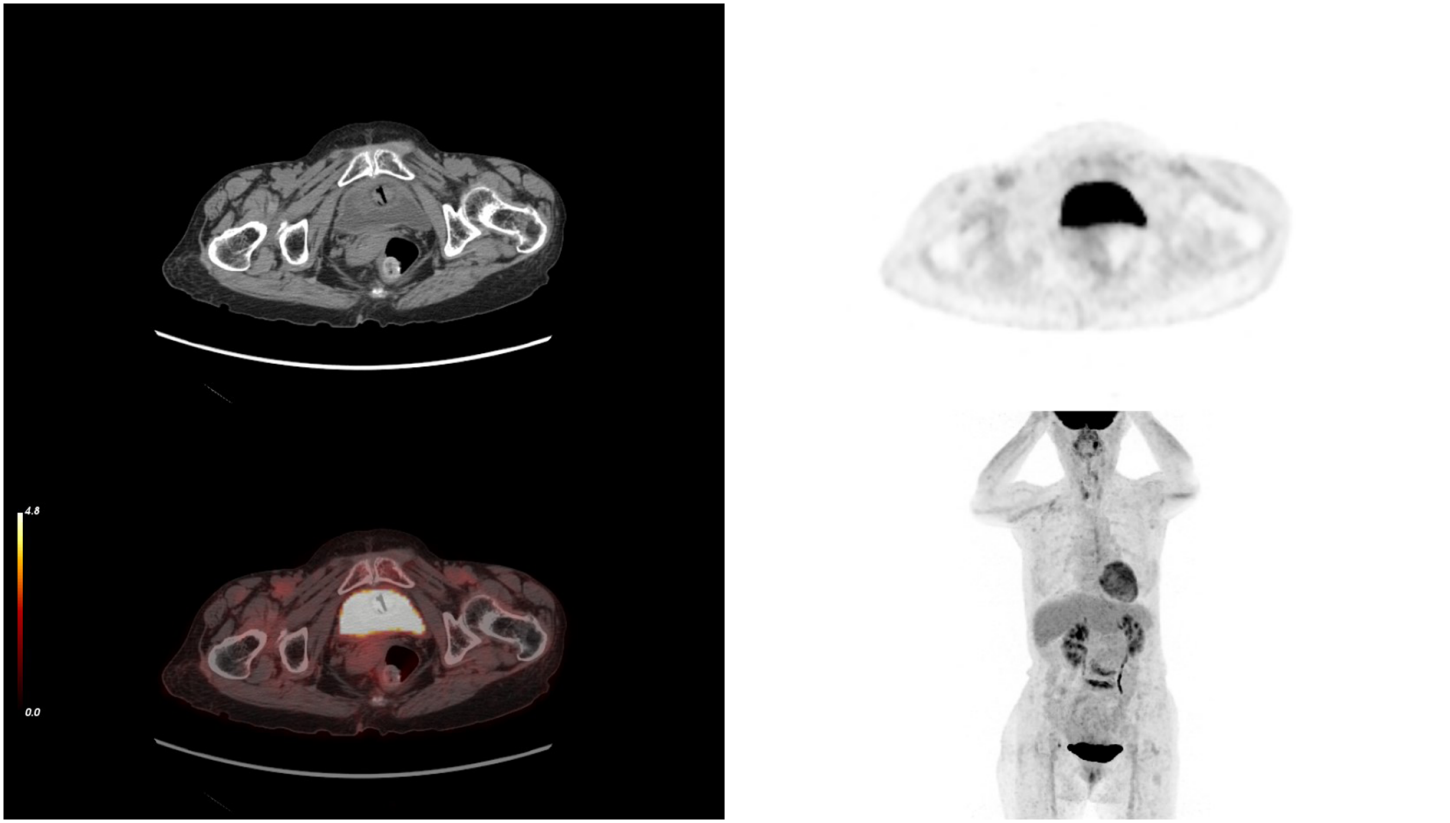
After 10 cycles of treatment, PET-CT results showing the complete disappearance of the tumor. PET-CT, positron emission tomography/computed tomography.

**Figure 6:**
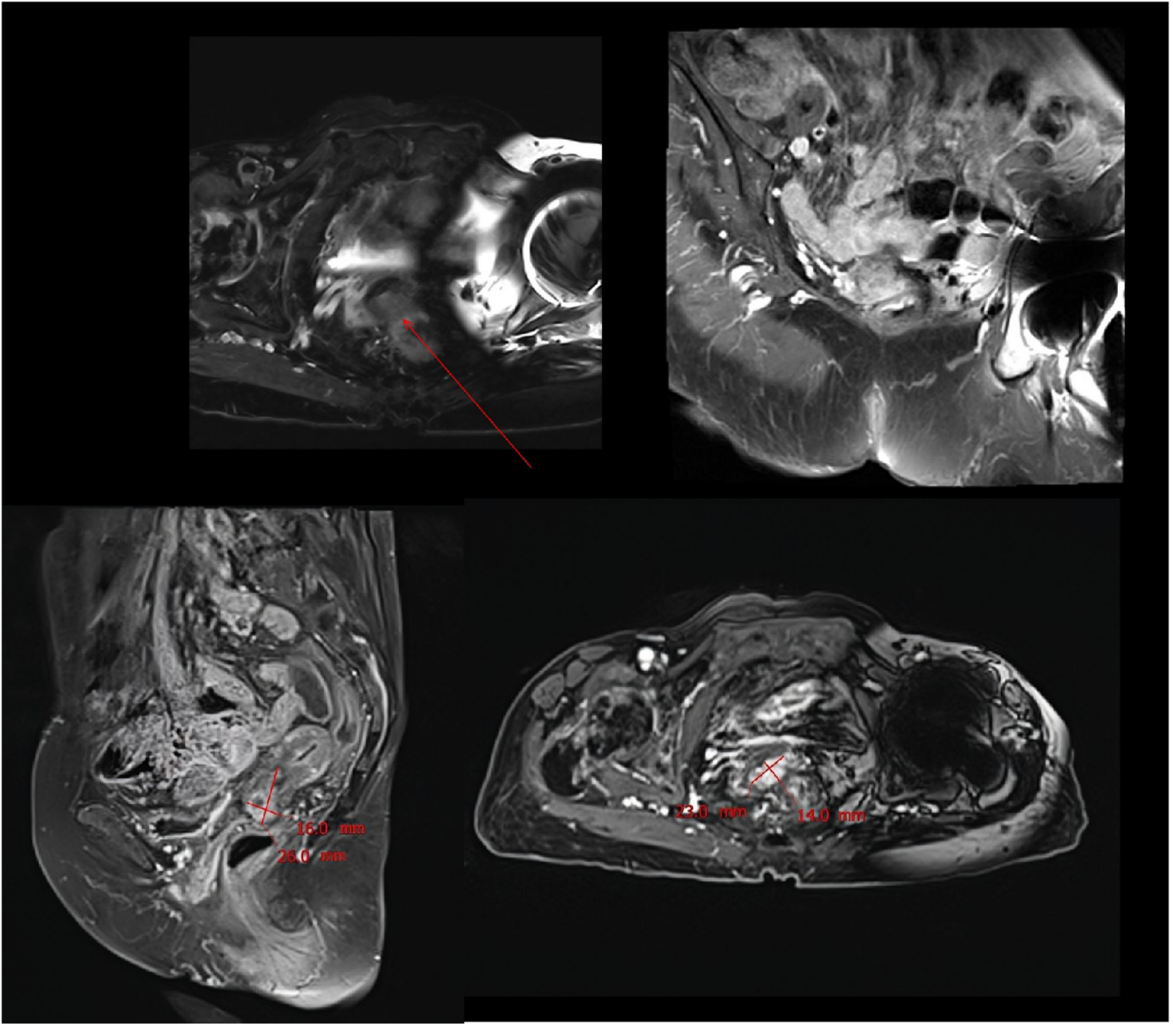
MRI scan performed after 13 months of combination therapy showed significant tumor recurrence.

SCC is extremely rare and has a poor prognosis in advanced stage patients, with no evidence of survival benefit from radiotherapy and chemotherapy. We advised the patient to undergo genetic testing to identify potential therapeutic targets that could inform a more personalized treatment approach. The genetic testing of the patient’s tumor tissue revealed the following mutations and characteristics (1): Somatic tumor mutations:An NRAS Q61K mutation was detected at a frequency of 6%;A BRAF G464E mutation was identified at a frequency of 6.9%;An ABL1 K609del mutation was identified at a frequency of 6.0%; A DNMT3A S129G mutation was identified at a frequency of 48.2%; An EPCAM A82G mutation was identified at a frequency of 48.8%; A PALB2 A38G mutation was identified at a frequency of 42.5% (2). The tumor’s Combined Positive Score (CPS) for PD-L1 expression was 45, indicating positive programmed death-ligand 1 (PD-L1) expression (**Figure 4**) (3). No genes associated with hyperprogressive disease (HPD) were found (4). No mutations were detected in either immunotherapy-positive associated genes (MMR-related genes, POLE, POLD1, DDR genes, KRAS, TP53) or immunotherapy-negative associated genes (B2M, DNMT3A, JAK1/2, ALK, ROS1, MET, VEGFA, PTEN, STK11) (5). The microsatellite instability (MSI) testing results indicate a microsatellite stable (MSS) status, with an MSI score of 0.0235 (values ≥0.4 classified as MSI-H, <0.4 as MSS). The CPS for PD-L1 expression indicates that the tumor is highly likely to be sensitive to ICIs, which could make immunotherapy a viable treatment option. Prior to initiating formal antitumor therapy, the patient underwent comprehensive biochemical testing. The baseline characteristics upon hospital admission are detailed in **Table 1**. As a result, the patient was administered a combination therapy consisting of bevacizumab (300 mg) plus pembrolizumab (200 mg) Q3W. After 2 cycles of the therapeutic regimen, the tumor shrank significantly, and the patient did not experience any side effects(**Figure 1B**). Since then, the patient has continued to receive the regimen and the tumor has continued to shrink. Ultimately, after completing 10 cycles of treatment with ICIs and anti-angiogenic drugs, the positron emission tomography/computed tomography (PET-CT) scan showed complete disappearance of the tumor, with no evidence of residual cancer in the body(**Figure 5**). Considering the patient’s advanced stage and the high malignancy of cervical sarcomatoid carcinoma, which is highly prone to recurrence, we continued with the original treatment plan for maintenance therapy. During the maintenance therapy period, the patient underwent regular follow-up evaluations without evidence of recurrence. Treatment was maintained for 13 months until MRI demonstrated recurrence (**Figure 6**). Subsequently, the patient was transferred to the Department of Radiotherapy in our hospital and began radiotherapy. We still conducted regular follow-ups for the patient. Given the extremely high malignancy of this rare tumor, although the tumor eventually recurred unfortunately, the combination therapy initially achieved a transient recurrence-free survival period. Moreover, no significant adverse reactions were observed during the drug treatment. Radiotherapy was discontinued after 3 months due to concurrent tumor progression observed during treatment. The patient is currently receiving palliative care only. Our data demonstrate a progression-free survival (PFS) exceeding 13 months and overall survival (OS) surpassing 22 months.

**Table 1:** Baseline characteristics of the patient.

Category	Details
Demographics	
Age/Sex	73-year-old, Female
Ethnicity	Han Chinese
ECOG Performance Status	1
Laboratory values	
HPV Status	Positive
p16 IHC	Positive
HPV DNA Typing Test	Positive for HPV type 70 (low-risk)
Albumin	34.0 g/L (Ref:40.0-55.0)
Alanine Aminotransferase (ALT)	11.6 U/L (Ref:7.0-40.0)
Aspartate Aminotransferase (AST)	14.7 U/L (Ref:13.0-35.0)
Serum creatinine (SCr)	39.0μmol/L (Ref:41-81)
Fasting Blood Glucose (FBG)	4.94 mmol/L (Ref:3.96-6.12)
C-reactive protein (CRP)	7.3 mg/L (Ref:0.0-6.0)
Erythrocyte Sedimentation Rate (ESR)	81.0 mm/h (Ref:0.0-20.0)
Carcinoembryonic Antigen (CEA)	6.2 ng/ml (Ref:0.0-5.0)

Notably, among all tumor markers monitored during treatment, only carcinoembryonic antigen (CEA) showed abnormal elevation, while other markers remained consistently within normal ranges. Although CEA levels were abnormally elevated during the clinical course, the fluctuations were relatively minimal (**Table 2**).

**Table 2:** Dynamic monitoring of CEA levels.

Time point	CEA level (ng/mL)	Normal range (ng/mL)	Tumor response evaluation
Pre-treatment baseline	6.2	0.0-5.0	Diagnosed with SCC
After 2 treatment cycles	4.2	0.0-5.0	PR
After 10 treatment cycles	5.3	0.0-5.0	PET-CT showed the complete disappearance of the tumor
After 13 treatment cycles	5.5	0.0-5.0	MRI detected tumor recurrence
Final follow-up (22 months)	7.0	0.0-5.0	Bone metastasis confirmed

## 3 Discussion

In our report, we detail a rare instance of SCC that was successfully treated and achieved a clinical cure, highlighting the effectiveness of pembrolizumab combined with bevacizumab treatment. To our knowledge, PubMed records as of July 2025 indicate fewer than 40 reported cases of cervical sarcomatoid carcinoma worldwide, consequently no treatment guidelines are currently available for this malignancy (1–7, 11). Typically, the management of the disease involves a combination of surgery, chemotherapy, and radiation therapy, based on the stage of the disease and the patient’s overall health status (6). At present, a standardized treatment protocol for SCC has not been established. In clinical practice, doctors will make professional judgments based on the main treatment methods for cervical cancer and sarcomatoid carcinoma. In the early stages of the disease, surgical treatment is given priority. For locally advanced cases that are resectable, a combination of surgery and chemoradiotherapy is used. For locally advanced cases that are unresectable and advanced-stage lesions, systemic drug treatment plans are adopted, with chemotherapy drugs for cervical cancer as the mainstay. While chemotherapy is frequently cited in the medical literature as the only class of therapeutic drugs, the overall prognosis for patients with this rare and aggressive form of cancer remains unfavorable (3, 6).

Because sarcomatoid carcinoma appears to be insensitive to radiotherapy and chemotherapy, and have limited therapeutic options, patients generally have a poor prognosis and a short survival time (2, 6, 11). Sarcomatoid carcinoma, despite limited literature, is described as an aggressive tumor with poor clinical outcome (12). The rarity of the disease complicates the development of standardized treatment protocols, and as such, treatment often relies on a combination of radiotherapy, surgery, and chemotherapy, tailored to the individual patient’s condition and disease stage (1, 6). Despite the advent of novel treatments such as immunotherapy and targeted therapy, which have revolutionized the management of various gynecological malignancies, there remains a conspicuous absence of targeted drug applications specifically for this rare tumor. This underscores the urgent need for research and clinical trials to identify effective, personalized treatment strategies for patients afflicted with SCC.

The PD-1 is one of the checkpoints that regulates the immune response. The expression of PD-1 on effector T-cells and PD-L1 on neoplastic cells enables tumor cells to evade anti-tumor immunity. Blockade of PD-1 is an important immunotherapeutic strategy for cancers. Pembrolizumab is a humanized monoclonal anti-PD-1 antibody that has been extensively investigated in numerous malignancies (9). Actually, the tumor microenvironment is a complex and interrelated environment, made up of various cell types such as endothelial cells, pericytes, immune cells, fibroblasts, and extracellularmatrix (13). Cancer cells manipulate their surrounding microenvironment by secreting extracellular signals that trigger tumor angiogenesis, boost cancer cell growth, and foster immune tolerance, thus evading detection by the immune system (14). Bevacizumab functions as a monoclonal antibody designed to target vascular endothelial growth factor A (VEGF-A), blocking its interaction with vascular endothelial growth factor receptor (VEGFR) and inhibiting angiogenesis, a process that supports tumor growth. As an early therapy aimed at the tumor microenvironment, incorporating bevacizumab into the standard treatment protocol introduces a new therapeutic strategy and provides an effective option for various advanced cancers that have a poor prognosis (10).

The growing availability of targeted drugs marks the advent of personalized medicine, while bevacizumab remains a mainstay in the treatment of various diseases. The collaboration of antiangiogenic therapy with ICIs can result in a synergistic therapeutic advantage (10, 14, 15). The synergistic antitumor effect of bevacizumab and pembrolizumab is primarily mediated through multi-level mechanisms (16). At the vascular level, VEGF blockade promotes tumor vascular normalization, improving vascular structure and function to facilitate immune cell infiltration (17). Regarding the tumor immune microenvironment, vascular normalization significantly enhances effector T cell infiltration while reducing the accumulation of various immunosuppressive cells, thereby effectively ameliorating the immunosuppressive state (18). On the metabolic level, the improved hypoxic condition alleviates the acidic tumor microenvironment and restores T cell function (19). Furthermore, VEGF inhibition downregulates immune checkpoint molecule expression and enhances T cell activation signaling pathways (20). A clinical study conducted in cervical cancer has confirmed that this combination regimen significantly improves the treatment response rate, demonstrating promising clinical translation potential (21). The combination therapy with pembrolizumab and bevacizumab has shown a synergistic effect in this patient, resulting in a significant tumor reduction without any adverse side effects. Meanwhile, anti-PD-1 immunotherapy activates immune cells, increases the secretion of IFN-γ, reduces the amount of VEGF, and thereby promotes vascular normalization (14). Hence, the marriage of antiangiogenic therapy with immunotherapy can interact to enhance the treatment’s effectiveness on tumor cells.

Considering that the tumor was not sensitive to chemoradiotherapy, our patient in this case was in a locally advanced stage with a high recurrence and metastasis rate, along with the patient’s advanced age and frailty, and the positive PD-L1 expression indicated by genetic testing, we opted for a treatment regimen similar to that used for pulmonary sarcomatoid cancer (22). The treatment involved a combination of pembrolizumab and bevacizumab.

**Figure 7:**
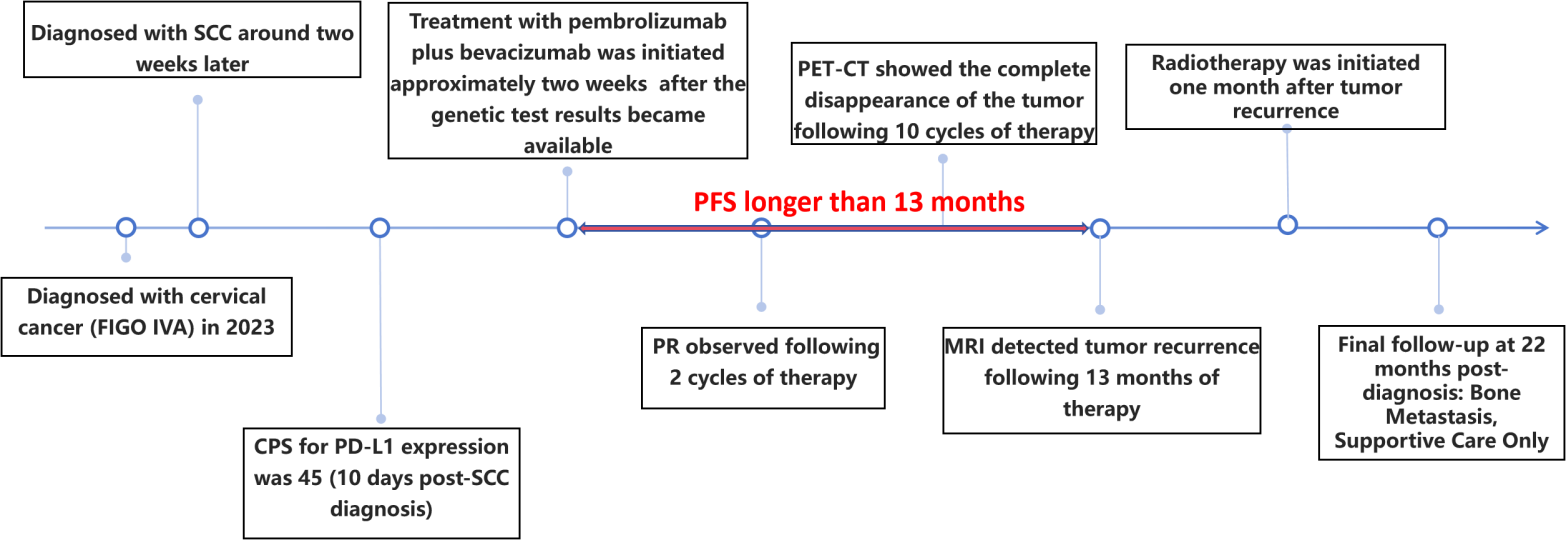
An overview of the patient’s entire treatment process.

The elderly female patient we admitted underwent 10 cycles of treatment with ICIs and anti-angiogenic drugs. Miraculously, the PET-CT scan data highlighted that the tumor had completely vanished, with no signs of cancer found throughout her body (**Figures 5**, **7**). Considering the high malignancy of SCC, which is highly prone to recurrence, we continued with the original treatment plan for maintenance therapy.

During the maintenance therapy period, the patient received regular follow-up evaluations, with no signs of recurrence detected. This condition persisted until 13 months after combination therapy, when MRI indicated tumor recurrence. (**Figure 6**). Since this disease is a rare tumor with no established treatment guidelines available, we referred to the management guidelines for cervical cancer and consulted a radiation oncologist for evaluation (23). Considering the patient’s advanced age and frail condition, the decision was made to refer her to the Department of Radiotherapy for the initiation of radiotherapy. The patient was then followed up regularly. Despite the eventual recurrence, we still consider this case a success worthy of reporting, as the patient achieved a PFS of over 13 months and an OS of over 22 months with the first-line combined treatment regimen. The success of this case suggests that for elderly and frail patients, traditional chemotherapy and radiotherapy may pose significant risks and side effects. In such scenarios, targeted therapy, being a relatively milder treatment modality, could potentially be a more suitable option for administering the care for these individuals.

The application of targeted therapy, which is designed to specifically attack cancer cells while sparing healthy ones, offers a less aggressive alternative to conventional treatments. This approach may be particularly beneficial for patients who may not tolerate the harsher effects of chemotherapy and radiation. By honing in on the molecular and cellular pathways that cancer cells rely on for growth and survival, targeted therapies like pembrolizumab and bevacizumab have shown promise in reducing tumor size and extending survival without the severe side effects often associated with more traditional cancer treatments.

## 4 Conclusion

Here we present an exceptionally rare case of SCC (initially staged as FIGO IV4A) exhibiting both low-risk HPV70 infection and p16 overexpression. The successful treatment of our patients demonstrates the efficacy and safety of targeted drugs applied in this rare tumor. Our case report confirms the therapeutic effectiveness of using ICIs in conjunction with antiangiogenic drugs to treat this PD-L1-positive and vascular-rich advanced rare tumor, providing evidence to support the clinical treatment of SCC. It also suggests that in rare tumors that are not sensitive to radiotherapy and chemotherapy, it is necessary to conduct genetic testing on tumor tissues to find effective therapeutic targets, at least the expression of PD-L1 is very meaningful. For elderly and frail patients with advanced tumors, blind pursuit of chemotherapy may not necessarily benefit patients, and may even shorten the survival period of patients. In the absence of sufficient chemotherapy evidence, seeking targeted therapy is a good strategy.

## Data Availability

The corresponding author can provide the datasets used or analyzed in this study upon receiving a reasonable request. The next-generation sequencing (NGS) data for this case report have been deposited into CNGB Sequence Archive (CNSA) of China National GeneBank DataBase (CNGBdb) with accession number CNP0006801.
